# Degree of Resection and Ki-67 Labeling Index for Recurring Meningiomas

**DOI:** 10.7759/cureus.1820

**Published:** 2017-11-03

**Authors:** Richard Menger, David E Connor Jr., Alvin Y Chan, Gary Jain, Anil Nanda

**Affiliations:** 1 Department of Neurosurgery, LSU Health Sciences Center Shreveport; 2 Medical College of Wisconsin; 3 Department of Surgery, University of Illinois Mt. Sinai

**Keywords:** meningioma, ki-67 labeling index, recurrence

## Abstract

Objective

Meningioma recurrence after resection is likely influenced by multiple surgical and histologic factors. In this study, the degree of resection and tumor immunoreactivity to MIB-1 (i.e., Ki-67 labeling index (LI)) are described in recurrent and non-recurrent meningioma cases.

Methods

Data regarding tumor location, the degree of resection, histologic features, and the degree of Ki-67 positivity were collected for 32 patients treated between September 2008 and July 2009. Follow-up for recurrence was assessed through five years.

Results

A total of 32 patients (13 males; 19 females) underwent resection. The mean age was 53.3 years. Gross total resection (GTR) occurred in 25 (78.1%) cases. Near-total resection (NTR) occurred in five (15.6%) cases. Subtotal resection (STR) occurred in two (6.2%) cases. The overall mean Ki-67 LI score was 9.75% (ranging between 1% to 48%). The mean Ki-67 LI for GTR, NTR, and STR cases were 8.0%, 10.2%, and 29.5% respectively. Tumor recurrence occurred in five (15.6%) patients. The mean Ki-67 LI for recurrence lesions was 22.2%.

Conclusion

We present our descriptive data for Ki-67 LI for initial tumors and recurrence. The risk of recurrence following resection of meningiomas may be associated with the degree of Ki-67 positivity.

## Introduction

The published rates of recurrence following resection of intracranial meningiomas range between 2% to 80% [1-12]. The factors affecting the incidence of recurrence have traditionally focused on operative variables, such as the degree of resection, age, and gender [1, 6, 13]. For example, Simpson established a grading system for the degree of resection and correlated each grade to a relative recurrence risk [9]. Thus, theoretically, certain histopathological and anatomical variations lead to a higher probability of recurrence. There is some evidence that the Ki-67 protein, a proliferative marker, could be an accurate predictor of recurrence, though the data is not conclusive [2].

We present our institution’s (Louisiana State University in Shreveport, Louisiana) data for the immunoreactivity to MIB-1 or Ki-67 labeling index (LI) for the recurrence of meningioma. We also provide a brief review of the literature.

## Materials and methods

The data for 32 patients who were operated on at our institution between September 2008 to July 2009 for intracranial or intraspinal meningioma were reviewed and then subsequently followed for five years. Patient charts, laboratory records, and radiographic imaging were analyzed. Data regarding the clinical course, recurrence rates, and the need for revision were collected over a five-year follow-up period. The original formalin-preserved specimens were obtained for computer-assisted quantitative immunohistochemical analysis.

The specimens were reviewed to determine the histologic World Health Organization (WHO) grade, the presence of cellular pleomorphism or tumor necrosis, progesterone receptor (PR) positivity, and immunoreactivity to MIB-1. For each case, formalin-fixed and paraffin-embedded specimens were sectioned at 4 μm and processed in an automated stainer, Ventana XT (Ventana Medical Systems, Tucson, AZ, USA), where deparaffinization and mild antigen retrieval occurred. The specimens were incubated with Ki-67 (clone 30-9) for 24 minutes. Biotinylated secondary antibodies were detected using the streptavidin/horseradish peroxidase method with diaminobenzidine as the chromogen. The slides were washed with ultraWash and hematoxylin counterstained. The percentage of immunopositive tumor cell nuclei was determined from digital images at 40x high power field (HPF) in the highest labeling regions of each case using an Automated Cellular Imaging System (ACIS) (ChromaVision Medical Systems, Inc., San Juan Capistrano, CA, USA).

Pre- and postoperative contrast-enhanced images were reviewed to identify the location and assess the resection degree. The grading was gross total resection (GTR) when there was no clear residual, near-total resection (NTR) when there was very minimal residual contrast-enhancement (including dural enhancement), and subtotal resection (STR) when there was substantial residual.

## Results

There were 13 males (40.6%) and 19 females (59.4%). The mean age at presentation was 53.3 years (ranging between 17-83). The majority of resected lesions were supratentorial, with the most common locations occurring in the anterior skull base (28.1%) and in proximity to the sagittal sinus (21.9%). The anatomic location is summarized in Table 1.

**Table 1:** Distribution of resected meningiomas by anatomic location

Location	Patient (Percentage)
Anterior Skull Base	9 (28.1%)
Parasagittal	7 (21.9%)
Convexity	6 (18.8%)
Petroclival	3 (9.4%)
Posterior Fossa	2 (6.3%)
Sphenoid Wing	2 (6.3%)
Cavernous Sinus	1 (3.1%)
Thoracic	1 (3.1%)
Cervical	1 (3.1%)
Total	32 (100%)

The pathology evaluation and the mean Ki-67 LI values are summarized in Table 2.

**Table 2:** Comparison of resected meningiomas by histologic grade and degree of Ki-67 labeling index (LI)

	WHO Grade	Patients	Mean LI
Primary Tumors	I	29	8.0
	II	3	27.0
	III	0	-
Recurrence	I	4	21.2
	II	1	26.0

Grade I lesion variants included 21 syncytial, three psammomatous, two translational, two cellular, and one microcystic meningiomas. Grade II lesions included two atypical and one clear cell meningioma. Tumor recurrence was identified to occur in five patients, four of whom required subsequent operative intervention. Recurrences were identified at a median of 34.1 months after initial resection. The mean Ki-67 LI for the initial resection samples of tumors that recurred was 22.2% (ranging between 11% to 48%) with a mean PR positivity of 52.4% (ranging between 0% to 92%). The degree of resection and the mean Ki-67 LI are summarized in Table 3.

**Table 3:** Comparison between the degree of operative resection of meningiomas and the mean degree of Ki-67 labeling index (LI)

Degree of Resection	Patient Number	Ki-67 LI
Gross Total Resection	25	8.7
Near Total Resection	5	7.0
Subtotal Resection	2	29.5

## Discussion

We present our Ki-67 labeling index data for initial resection of meningioma and recurrence. Meningiomas represent 13% to 26% of primary intracranial neoplasms, with the majority classified as WHO grade I or benign [14]. The recurrence of completely resected benign meningiomas ranges from 7% to 32%, increasing to 19% to 50% in higher Simpson grade resections [1, 4, 9, 15-16].

The proliferation markers associated with aggressive tumors in other organ systems (e.g., p53) have poor predictive value in meningiomas [17-22]. The MIB-1 monoclonal antibody recognizes Ki-67, a nuclear protein that is expressed during the G1, S, G2, and M phases of the cell cycles but absent in the G0 phase and early part of the G1 phase [23-25]. Consistent MIB-1 detection in tumor tissues makes it a better proliferation marker than proliferating cell nuclear antigen (PCNA) [26-27].

There is evidence that specific values of Ki-67 LI can help predict meningioma recurrence. Ki-67 LI is typically higher in recurrent tumors when compared to nonrecurrent tumors [2, 11, 27-29]. Kolles, et al. found Ki-67 LI to be a consistent factor for distinguishing anaplastic from benign meningiomas [29]. Bruna, et al. showed that Ki-67 LI was the only independent predictor of tumor recurrence and overall survival [2]. Perry, et al. showed that recurrence was more likely with a Ki-67 LI higher than or equal to 4.2% in GTR [20]. A similar review of 83 patients with a 10-year follow-up identified a threshold Ki-67 LI value of 10% [24]. Specifically, 30/31 patients with Ki-67 LI greater than or equal to 10% developed a recurrent tumor within 10 years and 100% of patients with Ki-67 LI lower than 10% were recurrence-free. Despite these results, laboratory differences in staining and counting methodologies make generalized application of a specific threshold difficult.

Several studies showed no correlation between Ki-67 LI and recurrence. In a review of 85 cases where 80% of the cases had recurrence, showed no significant difference in Ki-67 LI between the recurrent and nonrecurrent tumors [16]. A limitation was that regions within the specimens were chosen at random for Ki-67 LI scoring. Abramovich, et al. recommended that the highest degree of positive staining should be used because it represents the proliferative potential [30]. In a review of 169 completely resected WHO grade I meningiomas, Roser, et al. found no significant difference in survival based on the mean Ki-67 LI [15]. Some believe that higher Ki-67 LI in the case of higher grade tumors is more of a reflection of their anaplastic or malignant nature and that this testing should be reserved for cases with more borderline histologic features where classification and prognosis are more ambiguous [15, 19, 21, 30].

## Conclusions

Here we presented our data for Ki-67 LI for initial meningiomas and recurrence up to five years. The risk of recurrence following resection of meningiomas may be associated with the degree of Ki-67 positivity but further research is required to conclusively determine the true relationship.
